# Specific inhibition of ADAM17/TACE promotes neurogenesis in the injured motor cortex

**DOI:** 10.1038/s41419-018-0913-2

**Published:** 2018-08-28

**Authors:** Noelia Geribaldi-Doldán, Manuel Carrasco, Maribel Murillo-Carretero, Samuel Domínguez-García, Francisco J. García-Cózar, Juan Pedro Muñoz-Miranda, Valme del Río-García, Cristina Verástegui, Carmen Castro

**Affiliations:** 1 Área de Fisiología, Facultad de Medicina and Instituto de Investigación e Innovación en Ciencias Biomédicas de la Provincia de Cádiz (INiBICA), Universidad de Cádiz, Cádiz, Spain; 2 Área de Inmunología, Facultad de Medicina and Instituto de Investigación e Innovación en Ciencias Biomédicas de la Provincia de Cádiz (INiBICA), Universidad de Cádiz, Cádiz, Spain; 3 Departamento de Anatomía, Facultad de Medicina and Instituto de Investigación e Innovación en Ciencias Biomédicas de la Provincia de Cádiz (INiBICA), Universidad de Cádiz, Cádiz, Spain

## Abstract

Brain injuries in the adult mammalian brain are accompanied by a fast neurogenic response inside neurogenic niches. However, this response does not contribute to the generation of new neurons within damaged tissues like the cerebral cortex, which are essentially non-neurogenic. This occurs because injuries create a hostile environment that favors gliogenesis. Overexpression and sequential activation of the ADAM17/TGFα/EGFR signaling cascade are crucial for the generation of this gliogenic/non-neurogenic environment. Here, we demonstrate that chronic local infusion of a general metalloprotease inhibitor in areas of traumatic cortical injury in adult mice moderately increased the number of neuroblasts around the lesion, by facilitating the survival of neuroblasts and undifferentiated progenitors, which had migrated to the perilesional area from the subventricular zone. Next, we generated a dominant-negative version of ADAM17 metalloprotease, consisting of a truncated protein containing only the pro-domain (ADAM17-Pro). Specific inhibition of ADAM17 activity by ADAM17-Pro overexpression increased the generation of new neurons in vitro. Local overexpression of ADAM17-Pro in injured cortex in vivo, mediated by lentiviral vectors, dramatically increased the number of neuroblasts observed at the lesion 14 days after injury. Those neuroblasts were able to differentiate into cholinergic and GABAergic neurons 28 days after injury. We conclude that ADAM17 is a putative target to develop new therapeutic tools for the treatment of traumatic brain injury.

## Introduction

Brain lesions of different etiology, including traumatic and cerebrovascular insults, result in extensive neuronal loss and may cause irreversible cognitive deficits, sensory-motor alterations, or even personality disturbances^1^. Although no effective treatment is currently available to ameliorate neuronal loss, therapeutic strategies aimed to promote endogenous neuronal replacement constitute promising alternatives.

Central nervous system (CNS) injuries activate neural stem cells within physiological neurogenic niches: the dentate gyrus of the hippocampus (DG)^2,3^ and the subventricular zone (SVZ)^4,5^, facilitating the production of undifferentiated neural progenitor cells (NPC) and neuroblasts, which eventually attempt to migrate towards the site of injury. Accumulating evidence suggest that recruitment of SVZ-derived NPC may not be the only mechanism through which new neurons originate in injured cortical areas. NPC residing in the cortex can proliferate and generate new neurons in response to cortical injury in rodents^6,7^. However, neuronal replacement in lesions is variable and very limited^2,3,8–14^, mainly because a gliogenic environment rapidly arises within the injured tissue, where the vast majority of NPC differentiate into glial cells^15^. These, together with activated astrocytes, microglial cells, oligodendrocyte progenitors, and fibroblasts^16,17^ contribute to the formation of the glial scar. Cells forming the glial scar secrete a complex extracellular matrix that prevents neuronal migration towards the injury^18–20^. In addition, microglial cells activated in response to inflammation^21^ secrete cytokines and other factors that impair neuronal survival^22,23^. The epidermal growth factor receptor (EGFR)-activated signaling pathway contributes to the generation of a gliogenic/non-neurogenic environment around the injured area^24,25^. EGFR and its ligand transforming growth factor alpha (TGFα) are overexpressed in brain lesions together with the metalloprotease ADAM17/TACE, which performs t`he shedding of membrane-anchored TGFα releasing the soluble factor, which activates the EGFR pathway and leads to glial differentiation of NPC^14^. Specific inhibition of ADAM17/TACE leads to neuronal differentiation in vitro, reducing the generation of glial cells^14^.

We show in here that inhibition of metalloprotease activity in mechanical lesions of the adult-mouse primary motor cortex facilitates neurogenesis within the lesion, by promoting the migration and survival of neuroblasts from neurogenic regions and by inducing the differentiation of NPC towards the neuronal lineage. In addition, we show that specific inhibition of ADAM17 by overexpression of its pro-domain region^26,27^ highly promotes the generation of new cholinergic neurons within this type of lesions.

## Materials and methods

### Reagents

GM6001 (N-[(2R)-2-(hydroxami-docarbonylmethyl)-4-methylpentanoy]-L-tryptophan methylamide), the broad spectrum matrix metalloprotease inhibitor, was purchased from Calbiochem (San Diego, CA, USA), dissolved in dimethyl sulfoxide (DMSO) and diluted to a final concentration of 50 μM with sterile phosphate-buffered saline (PBS) prior to animal administration. Restriction enzymes were from Takara (Kusatsu, Japan). Other products, unless otherwise indicated, including custom-designed primers, were purchased from Sigma-Aldrich (St. Louis, MO, USA).

### Animal subjects

Two-month-old adult male CD1 mice were used for in vivo experiments. Seven-day postnatal (P7) CD1 mice were used for the isolation of NPC from the SVZ. Animals were housed under controlled conditions of temperature (21–23 °C) and light (LD 12:12) with free access to food (AO4 standard maintenance diet; SAFE, Épinay-sur-Orge, France), and water. Care and handling of animals were performed according to the Guidelines of the European Union Council (2010/63/EU), and following the Spanish regulations (65/2012 and RD53/2013) for the use of laboratory animals.

### SVZ cell isolation and culture

NPC were obtained from the SVZ of P7 mice following the same procedure described in ref. ^28^, and were cultured in defined medium (DM), composed of Dulbecco’s modified Eagle’s medium/F-12 nutrient mixture (DMEM/F-12) plus 1 mg/L gentamicin, 200 mM glutamine, and the B27 supplement without vitamin A (Invitrogen; Carlsbad, CA, USA). Epidermal growth factor (EGF, 20 ng/ml) and basic fibroblast growth factor (bFGF, 10 ng/ml), both from PeproTech (Frankfurt, Germany), were added to DM for NPC culture expansion in the form of neurospheres, but were withdrawn from the media for NPC differentiation experiments. Culture media and reagents, unless otherwise indicated, were from GIBCO (www.thermofisher.com/gibco).

### Cloning of mouse ADAM17 cDNA and production of the ADAM17-Pro mutant by site-directed mutagenesis

Total RNA was obtained from SVZ-derived NPC cultures and ADAM17 cDNA was amplified by RT-PCR and cloned into the pcDNA 3.3 plasmid, using the pcDNA 3.3 TOPO-TA cloning kit (Invitrogen; Carlsbad, CA, USA). Clones were analyzed by sequencing (Secugen; Madrid, Spain). One of the clones was chosen to subsequently generate the ADAM17-Pro-pcDNA 3.3 construct. A truncated form of ADAM17 stopping at aa 227 was engineered by site-directed mutagenesis. Mutations were introduced with the Site-Directed Mutagenesis Kit (*Stratagene*, Agilent Technologies; Santa Clara, CA, USA) and the following 5′-phosphorylated primers: Fw, 5′-GTG CTT CCA GGA GCG CAGC; Rw, 5′-TTT ACA AGT ATT CTT CAA GGG GTT AGG TTC AGC. The resulting plasmid was confirmed by sequencing (Secugen; Madrid, Spain).

### Generation of a lentiviral vector for in vivo expression of the ADAM17-Pro construct

ADAM17-Pro was directionally subcloned into a p-ENTR/D-TOPO vector and transferred by means of a Gateway LR ClonaseTM II Enzyme Mix system (Invitrogen, CA, USA), to a lentiviral shuttle vector containing a ZS-Green cassette, whose translation was governed by an internal recognition sequence (IRES) facilitating the independent co-expression of the two proteins driven by a CMV promoter. The resulting construct, pLenti-ADAM17-Pro-IRES-ZS-Green, was confirmed by sequencing (Secugen, Madrid, Spain).

To generate lentiviral particles, HEK293 cells were co-transfected with pLenti-ADAM17-Pro-IRES-ZS-Green, plus the pCMV-ΔR8.9, and pCMV-VSV-G vectors, necessary to produce the viral envelope and packaging. Transfection was performed in OPTI-MEM medium (GIBCO; www.thermofisher.com/gibco) using polyethylenimine. One hour later, this medium was substituted for DMEM supplemented with 10% fetal calf serum (GIBCO; www.thermofisher.com/gibco). Viral supernatants were collected from HEK293 cultures 48 and 72 h after transfection, and incubated for 30 min with the Lenti-X concentrator reagent (Clontech; Mountain View, CA, USA) at 4 °C; after a 50 min centrifugation (1500×*g*), the pellet was resuspended in 1 ml of PBS and treated with Lenti-X one more time. The resulting viral particles were capable of infecting mammalian cells, which consequently expressed ADAM17-Pro and the green fluorescent protein (GFP) ZS-Green as two independent proteins. Control cells, transduced with “empty” lentiviral particles lacking the ADAM17-Pro sequence, expressed only ZS-Green and, thus, accounted for the effects caused by the transduction procedure.

To measure viral titers, Jurkat cells were infected and the expression of ZS-Green was analyzed using a Cytoflex™ flow cytometer (Beckman, Indianapolis, IN) 48 h after transduction. Cell fluorescence was analyzed, and only viral preparations infecting more than 80% of cells were used. The viral titer of the lentivirus solutions was around 40×10^3^ TU/ml for both constructs. The presence of ADAM17-Pro mRNA in infected cells was determined by total RNA isolation, reverse transcription, and PCR amplification using a pair of primers (Fw, 5′-GGG CAG AAT ATA TAA CGT AGA GCC; Rw, 5′-AGG ACT GTT CCT ATC ACT GCA CT) that produced either a 1000-bp amplicon when hybridizing with the wild-type endogenous ADAM17 or, alternatively, a 400-bp amplicon when hybridizing with the mutant ADAM17-Pro. This method allowed us to detect mutant ADAM17-Pro mRNA even in cells that endogenously expressed native ADAM17.

### NPC transfection, differentiation, and immunocytochemistry

Neurosphere cells were disaggregated and adhered onto poly-L-ornithine-coated 1.8-mm-diameter round coverslips, in DM media without growth factors. Four hours later, cells were transfected with pcDNA3.3 plasmids containing the coding sequences for the expression of GFP, an ADAM17-GFP chimera, or an ADAM17-Pro-GFP chimera. Lipofectamine 2000 (Invitrogen; Carlsbad, CA, USA) was used for transfection of these plasmids. Cells were allowed to differentiate for 72 h, with a medium change after the first 24 h to eliminate Lipofectamine. Then, cells were fixed with 4% paraformaldehyde (PFA) and processed for βIII-tubulin immunodetection as previously described^28^. Antibodies used were: mouse anti-βIII-tubulin (1:1000; Cell Signaling Technology, Boston, MA, USA) and goat anti-mouse IgG labeled with AlexaFluor 594 (1:5000; Invitrogen, Carlsbad, CA, USA). Total nuclei were counterstained for 10 min with 0.1 mg/L DAPI. Transfected cells were identified by GFP auto-fluorescence. Transfection efficiencies were similar with the three plasmids used. Cells positive for βIII-tubulin and GFP were counted under a BX60 epifluorescence microscope (Olympus, Hamburg, Germany), and were expressed as percentage of transfected cells. Quantification was performed in 12 predetermined visual fields per coverslip. Experiments were repeated 3 times with triplicate samples, and results were expressed as the mean ± S.E.M.

### Mechanical lesions in brain cortex

Unilateral lesions were performed in the right brain cortex of adult mice anesthetized with an intraperitoneal injection of a 100 mg/kg ketamine and 20 mg/kg xylazine cocktail. Animals were placed in a stereotaxic frame (Kopf Instruments), and a small craniotomy was performed at +1.4 mm rostral and +1.5 mm lateral to Bregma. A controlled mechanical lesion was performed in the underlying primary motor cortex, using a manually driven drill (0.7 mm diameter) that was allowed to penetrate 1 mm below the bone surface.

### Studies describing neurogenic responses in mechanical lesions

In order to study the time course of neurogenesis and gliogenesis in mechanical lesions, as well as in the SVZ and the DG of injured brains, mice were injured using the procedure mentioned above, and were sacrificed at 3, 7, or 14 days post - injury (dpi). Mice were given three intraperitoneal injections of BrdU (70 mg/kg each) separated by 3-h intervals either 1 day before sacrifice (in the 3 dpi group) or the same day of sacrifice (7 and 14 dpi groups). Mice were sacrificed by brain perfusion, and brains were processed for post-mortem studies as described below.

### GM6001 infusions

In the same surgical acts in which cortical lesions were performed, the animals were prepared for chronic infusions of either GM6001 or vehicle, applied locally in the lesion. For this, Alzet osmotic mini-pumps (Charles River Spain; Barcelona, Spain, www.criver.com) were implanted subcutaneously in 24 animals and connected to infusion cannulas (brain kit II, Alzet) whose tips were placed 0.5 mm deep into the lesion, allowing a continuous delivering of either a 50-µM solution of GM6001 in PBS (containing 0.4% DMSO) or vehicle. Treatments lasted either for 14 dpi (Alzet 1002) or for 28 dpi (Alzet 1004). In addition, all these mice received three intraperitoneal injections (70  mg/kg each) of the thymidine analog bromodeoxyuridine (BrdU) on day 14. Each BrdU dose was separated from the next by a 3-h-time interval, and animals were sacrificed 3 h after the last BrdU dose within the same day.

Thus, brain studies were performed at two different time points: 14 and 28 dpi. The two groups of animals receiving a continuous treatment during 14 days (vehicle; *n* = 6 and GM6001-treated mice; *n* = 6) were sacrificed on day 14 and received three BrdU injections on the day of sacrifice. They were named as *c14 dpi* (letter c indicates continuous treatment; 14 indicates the duration of the treatment). The other two groups of animals receiving a continuous treatment during 28 days (vehicle; *n* = 6 and GM6001-treated mice; *n* = 6) were sacrificed on day 28, roughly 14 days after the three BrdU pulses administered on day 14. These were named as *c14*+*14 dpi* (letter c indicates continuous treatment; 14+14 indicates the duration of the treatment before and after BrdU administration).

In a different set of experiments, aimed to study the migration of progenitors from neurogenic regions towards the injured area, mice received BrdU injections 6, 5, and 4 days before the injury was performed. On the day of injury, animals were implanted osmotic minipumps to allow the continuous delivering of a 50-µM solution of GM6001 or vehicle, until they were sacrificed 14 dpi (see scheme in Fig. 3). This paradigm allowed for substantial clearance of BrdU from the mouse body before performing the cortical lesions and, therefore, it allowed BrdU labeling of NPC in the SVZ but not locally in the injured tissue. These mice (vehicle; *n* = 6 and GM6001-treated; *n* = 6) were sacrificed on day 14 after injury and were named as *6*+*14 dpi*.

### Local lentiviral transductions

In a separate set of experiments, mice (*n* = 36) were mechanically injured in the cortex as explained before and were injected, locally in the lesion, with lentiviruses carrying either the dominant-negative ADAM17-Pro cDNA in combination with ZS-Green cDNA or the ZS-Green cDNA alone. mRNA expression was driven by a CMV promoter and coordinated synthesis of both proteins (ADAM17-Pro and ZsGreen) was controlled by an IRES element. The viral titer of the lentivirus solutions was 40 × 10^3^ TU/ml for both constructs. 1 μl of this lentivirus solution was injected at the lesion site to induce the expression, of ADAM17-Pro (the prodomain form of ADAM17; *n* = 12) and ZS-Green. Control mice were injected either with 1 µl of the “empty” (ZS-Green alone) lentiviral vector solution (which only induced the expression of ZS-Green in infected cells and accounted for any effect caused by transduction; *n* = 12) or with vehicle (PBS; *n* = 12). For local administration, a 10-µl Hamilton syringe (0.485-mm internal diameter) was placed in the stereotaxic frame and used to deliver 1 µl of the lentiviral preparations or vehicle into the lesion area, at a speed of 0.1 µl/min; afterwards, the syringe was left in place for other 10 min before removal.

After these local single injections, mice were divided further into two experimental groups. The first group received, on day 14 post-lesion, three intraperitoneal injections of BrdU (70 mg/kg each) separated by 3-h intervals, and were sacrificed 3 h after the last BrdU dose within the same day This group was named as *s14 dpi* (letter s indicates single injection and 14 indicates the days post injection and injury). The other experimental group of mice received a single BrdU pulse every 2 days during the 2 weeks that followed the surgical procedure, and were sacrificed 14 days after the last BrdU injection. These are referred to as *s14*+*14 dpi* (letter s indicates single injection and 14+14 indicates the dpi/injection before and after BrdU administration).

### Brain processing and immunohistochemistry

Brain removal, processing, sectioning, and immunohistochemical detection of BrdU and other cell markers were performed as previously described^28,29^. Primary antibodies used were: mouse monoclonal anti-BrdU (1:100) and rabbit polyclonal anti-GFAP (1:3000) from Dako (Glostrup, Denmark); rat monoclonal anti-BrdU (1:100) from Abcam (Cambridge, UK); goat polyclonal anti-doublecortin (1:200) and goat polyclonal anti-nestin (1:200) from Santa Cruz Biotechnology (Santa Cruz, CA, USA); mouse monoclonal anti-NeuN (1:100), goat polyclonal anti-ChAT (1:100), and mouse monoclonal anti-parvalbumin (1:100) from Merk Millipore (Billerica, MA, USA), and mouse monoclonal anti-VGLUT2 (1:100) from Abcam (Cambridge, UK). Secondary fluorescent antibodies used (1:1000) were all from Invitrogen (Carlsbad, CA, USA): donkey anti-rabbit IgG conjugated to AlexaFluor 594 or AlexaFluor 488; donkey anti-mouse IgG conjugated to AlexaFluor 405 or AlexaFluor 488; donkey anti-goat conjugated to AlexaFluor 594; and donkey anti-rat conjugated to AlexaFluor 488 or AlexaFluor 594. Biotinylated donkey anti-goat IgG (1:250) was from Sigma-Aldrich.

### Stereology and quantitative analyses

Stereological methods for unbiased cell counting were used to estimate the number of cells positive for the markers analyzed^30,31^ and 5–6 animals were used per condition. See supplementary material for more detailed information.

In the case of the neurogenic regions SVZ and DG, ipsilateral and contralateral sides of the brain (in relation to the cortical lesion) were counted separately. Quantifications were done in 1 out of every five 30-μm-thick serial coronal sections spanning, in the rostrocaudal axis in relation to Bregma, from +1.54 to −0.94 mm for the SVZ, and from −0.94 to −3.64 mm for the DG^32^. In each section, cell number was counted throughout the entire lateral walls of the lateral ventricles (for the SVZ), or the entire DG. The absolute number of cells positive for a given marker in these structures was defined as: *N* = Ʃ*Q* x 1/*ssf*, where Ʃ*Q* is the total number of cells counted in all the sections for that structure and *ssf* is the fraction of sections counted (1/5).

To analyze cell number in the lesion perimeter, 3–5 sections containing the cortical injury were selected per mice. In each section, cells positive for the desired markers were quantified within a 200-μm-wide band of tissue adjacent to the lesion border, and the area occupied by this tissue band was also measured using the ImageJ software. Cell density (number of cells counted divided by lesion area and by section thickness) was calculated for each section, and averaged for each animal. Inter-animal differences in perilesional areas were minimal (30 ± 4 × 10^3^ μm^2^/section).

### Statistical analysis

When more than two treatment groups were compared, statistical analyses were performed using one-factor ANOVA, followed by post-hoc Bonferroni. The Student's *t* test was used when only one treatment group was compared with the control. When ipsilateral versus contralateral brain hemispheres were compared, the Student's *t* test for paired samples was used. The U-Mann Whitney test was used in the case of non-parametric distribution of samples. Differences were considered significant at values of *p* < 0.05.

## Results

### Description of the cortical injury model

We performed controlled mechanical injuries in adult mice that produced discrete lesions restricted to the primary motor cortex-gray matter. The time-course of the neurogenic response to the lesion was analyzed 3, 7, and 14 dpi in the SVZ and DG, as well as within the perilesional area. Mice were intraperitoneally injected with BrdU on the day of sacrifice. We analyzed the following cell markers: BrdU for proliferative cells; nestin for undifferentiated NPC; DCX for neuroblasts; GFAP for astrocytes; and NeuN for mature neurons.

### Neurogenic response to cortical injuries in the adult mouse brain

Proliferative (BrdU^+^) cells were found within the injured area as soon as 2–3 dpi (mice sacrificed on day 3 received BrdU on day 2, thus BrdU^+^ cells may have incorporated BrdU from days 2 to 3); the number of proliferative cells increased dramatically (5-fold) from 2–3 dpi to 7 dpi, decreasing to almost undetectable levels at 14 dpi (Fig. S1). The number of nestin^+^ undifferentiated progenitors was much higher than that of BrdU^+^ cells, but it showed a fluctuation pattern with time identical to that of BrdU^+^ cells (Fig. S2 A, C). In fact, the proportion of BrdU^+^ cells expressing nestin did not significantly change over time (Fig. S2 D). GFAP^+^ cells were not very abundant at 3 dpi, but increased dramatically on days 7 and 14 (Fig. S2 B, E); in fact, at 14 dpi, nearly 70% of BrdU^+^ cells expressed the astrocytic marker GFAP^+^ (Fig. S2 F), while no DCX^+^ neuroblasts were found in the injured area at any time point.

We also analyzed the concomitant response of physiological neurogenic niches to cortical injury. A proliferative reaction was found in both the ipsilateral SVZ (Fig. S3) and DG (Fig. S4) of injured mice, at all time points tested (2–3, 7, and 14 dpi). The response in the DG was stronger than in the SVZ (Fig. S3 and S4).

### Description of the paradigm used to study the effects of metalloprotease inhibition in mouse cortical lesions

According to previous reports^14^, inhibition of the ADAM17 metalloprotease in vitro increases neuronal differentiation from NPC. Thus, we hypothesized that local metalloprotease inactivation within cortical lesions could enhance the generation of new neurons from nestin^+^ precursor cells.

Metalloprotease activity was inhibited by osmotic minipumps that chronically released, during 14 or 28 days, either the broad metalloprotease inhibitor GM6001 (50 μM) or vehicle. All mice, regardless of treatment duration being 14 or 28 days, were injected with BrdU on day 14 to label proliferative cells. In mice treated with GM6001 or vehicle during the first 14 dpi (referred to as c14 dpi on the figures), we analyzed the number of BrdU^+^, nestin^+^, DCX^+^, Iba1^+^ cells, and the GFAP^+^ burden. In addition, in mice treated for 28 dpi, we analyzed whether cells labeled with BrdU on day 14 had survived, remained undifferentiated, or had differentiated into neuroblasts, neurons or glia on day 28 (this group is referred to as c14+14 dpi on the figures).

### Inhibition of metalloprotease activity in cortical lesions promotes the generation of undifferentiated progenitors

**Fig. 1:**
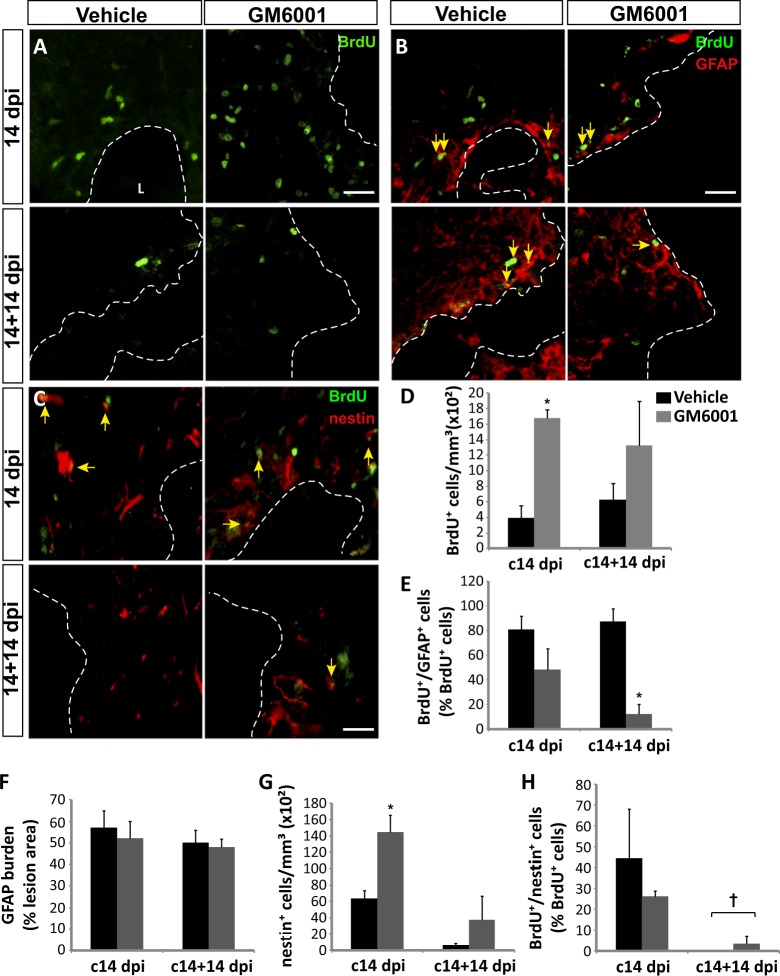
Local inhibition of metalloprotease activity in the injured cortex increases the number of neural progenitors and limits their glial differentiation. **a**–**c** Representative confocal (**a**, **b**) and fluorescence (**c**) microscopy images of the area surrounding cortical lesions in mice locally-infused with vehicle or the metalloprotease inhibitor GM6001 (50 μM). Mechanical cortical lesions were unilaterally performed in the primary motor cortex of adult mice, and minipumps were implanted to locally deliver GM6001 or vehicle for 14 or 28 days; at 14 days post-injury (dpi), all mice were intraperitoneally injected with BrdU to label proliferating cells. Mice were sacrificed either at the end of that day (c14 dpi groups) or 14 days later (c14+14 dpi groups). Dotted lines delineate cortical lesion borders. Arrows indicate cells double-labeled for BrdU/GFAP (**b**) or BrdU/nestin (**c**). Scale bar = 50 µm. **d** Quantification of BrdU^+^ cells/mm^3^ in the perilesional area of the indicated animal groups. **e** Graph shows the percentage of BrdU^+^ cells that coexpressed GFAP in the perilesional area. **f** Graph shows the percentage of perilesional area occupied by GFAP^+^ labeling (GFAP burden). **g** Quantification of nestin^+^ cells/mm^3^ in the perilesional area of the indicated animal groups. **h** Percentage of BrdU^+^ cells that coexpressed nestin in the perilesional area. Data shown are the mean ± S.E.M.; *n* = 3–6 animals per group. Statistical analysis: ANOVA and Bonferroni posttest; *p < 0.05 when compared to vehicle-treated mice; ^†^*p* < 0.05 when compared to the c14 dpi group. L lesion

In vehicle-treated mice, BrdU^+^ cells (Fig. 1a, d) were found around the damaged cortex at 14 dpi, together with nestin^+^ and GFAP^+^ cells (Fig. 1b, c, f, g). Treatment of lesions with GM6001 for 14 days resulted in a robust increment of BrdU^+^ (Fig. 1a, d) and nestin^+^ cells (Fig. 1c, g) around the lesion. Nearly 5% of these BrdU^+^ cells co-localized with the microglial marker Iba1, with no differences between control and treated animals (data not shown).

Animals labeled with BrdU at day 14 and analyzed at day 28 (c14+14 dpi) presented a large dispersion in the number of BrdU^+^ cells around the lesion. Nevertheless, BrdU counts at 28 dpi were similar to those found at 14 dpi (Fig. 1a, d), suggesting that most BrdU^+^ cells had survived for 14 days after BrdU labeling in both treated and non-treated lesions. Nestin^+^ cells were scarce in the perilesional area at 28 dpi (Fig. 1c, g), but the number of remaining cells co-expressing nestin and BrdU was slightly higher in GM6001-treated than in control lesions (Fig. 1c, h).

### Prolonged treatment with GM6001 reduces glial differentiation from NPC in cortical injuries

A strong overexpression of the astroglial marker GFAP occurred within the injured cortex at 14 and 28 dpi (Fig. 1b, f), with no differences in rough GFAP burden observed between control and GM6001-treated mice. This suggested that chronic administration of GM6001 did not significantly affect astrogliosis. However, to distinguish the newly-formed astrocytes, emerging from NPC, from reactive astrocytes within the lesion, we focused on the analysis of BrdU^+^ cells.

In control lesions, at 14 dpi, almost 80% of BrdU^+^ cells expressed GFAP (Fig. 1e), while more than 40% of BrdU^+^ cells expressed nestin (Fig. 1h), suggesting a partial overlap of nestin and GFAP expression in BrdU^+^ cells, in agreement with previous reports. At 28 dpi, 80% of BrdU^+^ cells were still GFAP^+^, while nestin expression was considerably reduced. These results indicated that the vast majority of BrdU^+^ cells had gradually differentiated into astrocytes within control lesions.

Interestingly, chronic administration of GM6001 for 14 or 28 days reduced the percentage of BrdU^+^ cells that expressed GFAP (Fig. 1b, e), and this reduction reached statistical significance at 28 dpi, when a dramatic decrease on newly-formed astrocytes was observed.

### Inhibition of metalloprotease activity in cortical lesions promotes the generation of neuroblasts and mature neurons

**Fig. 2:**
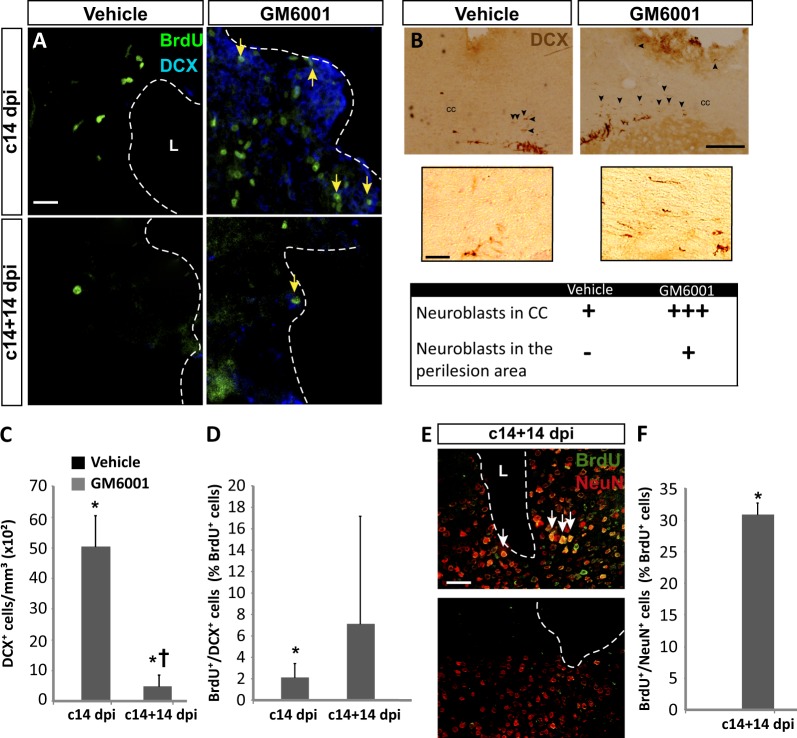
Local inhibition of metalloprotease activity in the injured cortex increases neuroblast number and promotes their differentiation to mature neurons. **a** Confocal representative images of the area surrounding cortical lesions in mice injured and treated as described in the legend of Fig. 1. **b** Light-microscopy photomicrographs show coronal sections of mice brains that have been treated with GM6001 or vehicle, as indicated, for 14 days; these sections, in which the cortical lesions and the SVZ dorsolateral corner can be observed, were immunostained for DCX using the peroxidase-diaminobenzidine method. The table below shows a semiquantitative estimation of the abundance of DCX^+^ cells in the indicated cerebral regions. Arrowheads indicate DCX^+^ cells. **c** Quantification of DCX^+^ cells/mm^3^ in the perilesional area of the indicated animal groups. **d** Percentage of BrdU^+^ cells that coexpressed DCX in the perilesional area. **e** Fluorescence representative images of the area surrounding cortical lesions. Arrows indicate cells double-labeled for BrdU and NeuN. **f** Percentage of BrdU^+^ cells that coexpress NeuN in the perilesional area. Data are the mean ± S.E.M.; *n* = 3–6 animals per group. Statistical analysis: ANOVA and Bonferroni posttest was used in (**c**) and (**d**) and Student´s *t* test was used in (**f**); *p < 0.05 when compared to vehicle-treated mice; ^†^*p* < 0.05 when compared to the c14 dpi group. Dotted lines delineate cortical lesion borders. Scale bars = 50 µm in (**a**, **e**) and lower panels of (**b**); 100 µm in upper panels of (**b**). CC corpus callosum, dpi days post-injury, L lesion

We next studied whether GM6001 had an effect on neuroblast formation within the injured cortex. As expected, DCX^+^ neuroblasts were absent in vehicle-treated animals at 14 and 28 dpi (Fig. 2a, c). Interestingly, in mice treated with GM6001, a significant number of DCX^+^ cells were found within the injured area at 14 (c14 dpi) and 28 dpi (c14+14 dpi), some of which co-localized with BrdU (Fig. 2a, d).

At the perimeter of GM6001-treated lesions, DCX^+^ neuroblasts showed a predominantly rounded morphology, with very little or no neurites, different from the bipolar shape of SVZ neuroblasts. In both control and GM6001-treated mice, DCX^+^ neuroblasts with a bipolar morphology were observed beyond the normal SVZ perimeter, crossing through the corpus callosum and forming a rough gradient towards the injury (Fig. 2b). However, these migrating cells were more abundant in GM6001-treated mice, and only these mice presented DCX^+^ cells at the site of injury (Fig. 2b).

The number of DCX^+^ cells found in GM6001-treated injuries at 14 dpi was significantly reduced at 28 dpi (Fig. 2c), inferring that part of the former neuroblasts found at 14 dpi might have differentiated into mature neurons at 28 dpi, losing DCX expression. To test this possibility, we analyzed the expression of the mature neuron marker NeuN in the c14+14 dpi group, and found that ∼30% of BrdU^+^ cells within GM6001-treated cortical injuries expressed NeuN (Fig. 2e, f).

From all the above results, we concluded that metalloprotease inhibition within the injured cortex favored the differentiation of NPC towards the neuronal, and not the glial, lineage.

### Metalloprotease inhibition allows the migration of cells from neurogenic regions towards the injured area

**Fig. 3:**
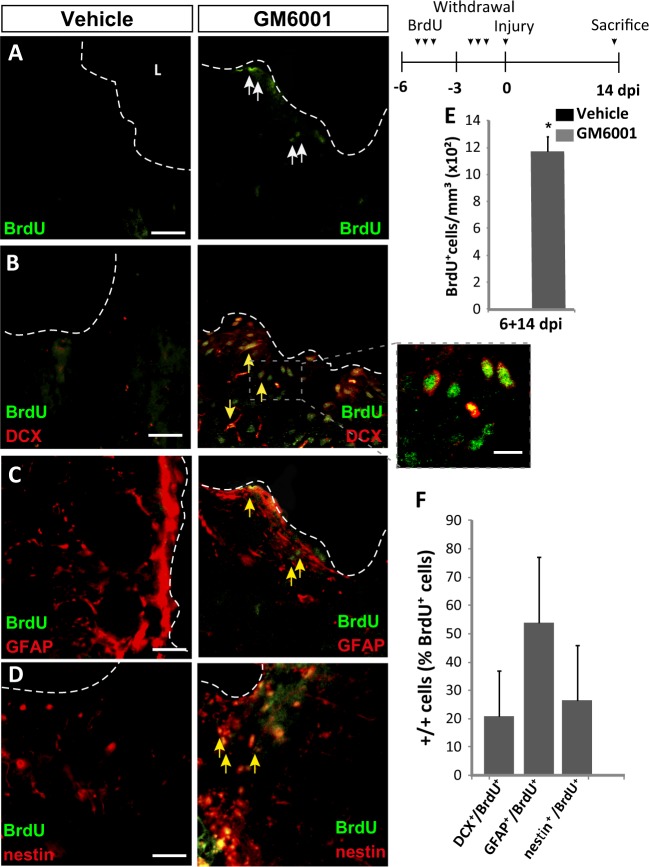
Local inhibition of metalloprotease activity in the injured cortex increases the migration of neural precursors from neurogenic regions to the damaged area. **a**–**d** Fluorescence representative microphotographs of the area surrounding cortical lesions of mice locally-infused with vehicle or the metalloprotease inhibitor GM6001. Dotted lines delineate cortical lesion borders, and arrows indicate double-labeled cells; scale bar = 50 µm. The upper right panel shows the experimental procedure followed to label proliferating neural precursors with BrdU exclusively in neurogenic niches and not locally in the lesion area: mice received peritoneal injections of BrdU on days 6, 5, and 4 before performing the cortical injury (days −6 to −4); then, we waited 3 more days to allow for complete withdrawal of BrdU from the animal organism (days −3 to 0); on day 0, cortical lesions and 14-day treatments with vehicle or GM6001 were done as described in the legend of Fig. 1. This group was named as 6+14 dpi. With this protocol, any BrdU^+^ neural cell found in the perilesional area in the 6+14 dpi group must have migrated from neurogenic niches. **e** Quantification of BrdU^+^ cells/mm^3^ in the perilesional area of the indicated animal groups. No BrdU^+^ cells were found in vehicle-infused animals. **f** Percentage of BrdU^+^ cells that coexpressed DCX, GFAP, or nestin in the perilesional area, as indicated. Data are the mean ± S.E.M.; *n* = 3–6 mice per group. Statistical analysis: Student's *t* test; *p < 0.05 when compared to vehicle-treated mice. dpi days post-injury, L lesion

NPC found within the injured cortical tissue could originate in neurogenic regions and reach the lesion after a process of migration or, alternatively, be generated following the activation of local neural stem cells. To determine which the case was, a group of mice received BrdU injections 6, 5, and 4 days before injury; this paradigm allowed for substantial elimination of BrdU from the mouse body before performing the cortical lesions. Using this BrdU administration protocol, only cells from physiological neurogenic niches could incorporate BrdU before injury; also, by not prolonging too much the time interval between the last BrdU injection and the injury, we could assure that labeled cells would still be close to the SVZ or, at most, halfway between the SVZ and the olfactory bulb. At the time of injury, osmotic minipumps were implanted to release either vehicle or 50 μM GM6001 locally at the site of injury. Animals were sacrificed at 14 dpi (the group is referred to as 6+14 dpi on the figures) and brains were processed to detect BrdU^+^ cells together with DCX, GFAP or nestin. In vehicle-treated mice, no BrdU^+^ cells were found at the site of injury, whereas mice treated with GM6001 showed a significant amount of BrdU^+^ nuclei, 52% of them being GFAP^+^, 28% nestin^+^, and 20% DCX^+^ (Fig. 3). These results indicated that, in the presence of GM6001, BrdU^+^ cells born in distant neurogenic niches (likely, the SVZ) reached the injured cortical tissue and survived.

### Metalloprotease inhibition increases the SVZ response to injury

**Fig. 4:**
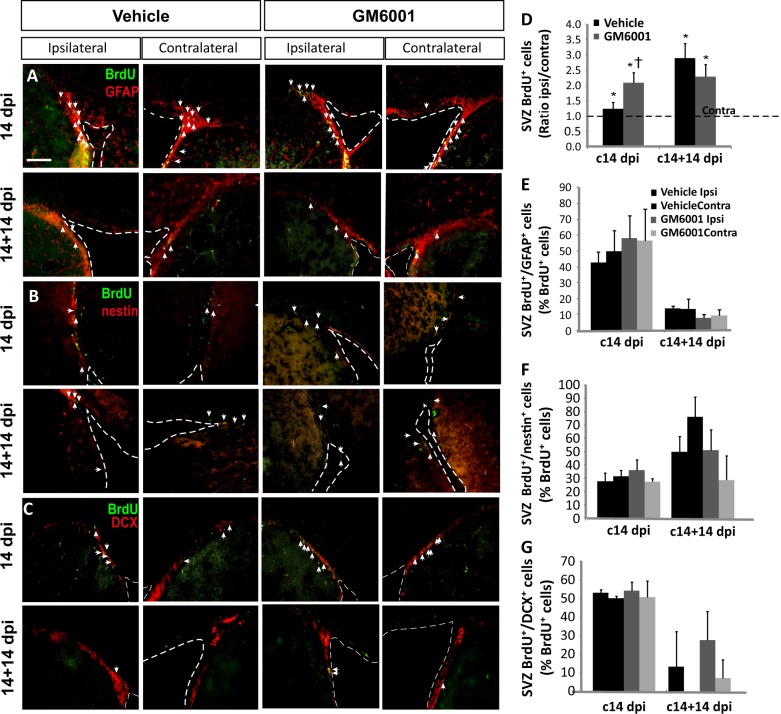
Metalloprotease inhibition modifies the neurogenic response of the SVZ to an injury. **a**–**c** Fluorescence microscopy images of the SVZ of adult mice bearing unilateral cortical lesions, in which either vehicle or the metalloprotease inhibitor GM6001 was locally infused. Cortical injuries, treatments, and BrdU injections were performed in adult mice cortex as described in the legend of Fig. 1. Dotted lines delineate lateral ventricle walls. Arrows indicate cells double-labeled for the corresponding pair of markers. Scale bar = 200 µm. **d** Graph represents the average ratios obtained when dividing the number of BrdU^+^ cells in ipsilateral SVZs by the number of BrdU^+^ cells in the corresponding contralateral SVZs. Statistical analysis: *p < 0.05 when comparing ipsilateral with contralateral SVZs in a Student’s *t* test for paired samples; ^†^*p* < 0.05 when comparing GM6001- with vehicle-injected mice of the c14 dpi group (Student’s *t* test for equal-variance unpaired samples). **e**–**g** Percentage of BrdU^+^ cells that coexpress: **e** GFAP, **f** nestin, or **g** DCX. Statistical analysis: ANOVA and Bonferroni posttest. Data are the mean ± S.E.M.; *n* = 3–6 animals per group. dpi days post-injury, SVZ subventricular zone

To determine the extent to which injury activated SVZ neurogenesis, and whether GM6001 treatment could modulate this activation, we studied neurogenesis in the SVZ of mice labeled with BrdU at 14 dpi and sacrificed at 14 or 28 dpi (c14 dpi and c14+14 dpi groups, respectively). We have calculated, for each animal, the ratio of ipsilateral BrdU^+^ cells to contralateral BrdU^+^ cells (Ratio ipsi/contra in the figures), as a measure of SVZ reaction to the injury. In all vehicle- and GM6001-treated mice from c14 and c14+14 groups, significantly greater numbers of BrdU^+^ cells were found in the ipsilateral SVZs than in the contralateral SVZs (Fig. 4d, asterisks). Furthermore, GM6001 treatment significantly increased the ratio of ipsilateral BrdU^+^ cells to contralateral BrdU^+^ cells in c14 mice (Fig. 4d). In contrast, neither GM6001 nor the lesion itself had any effect on the proportion of BrdU^+^ cells within the SVZ that expressed nestin, GFAP, or DCX (Fig. 4a–c, e–g).

### Overexpression of the ADAM17 pro-domain in NPC cultures promotes neuronal differentiation in vitro

Among candidate metalloproteases targeted by GM6001, we hypothesized that inhibition of ADAM17 was specifically mediating the above-mentioned effects, as previously suggested^33^. In search for a biomolecular tool designed to inhibit ADAM17 activity, we overexpressed the pro-domain of ADAM17 (ADAM17-Pro), regarded in previous studies as a potent inhibitor of this metalloprotease^26,27^.

**Fig. 5:**
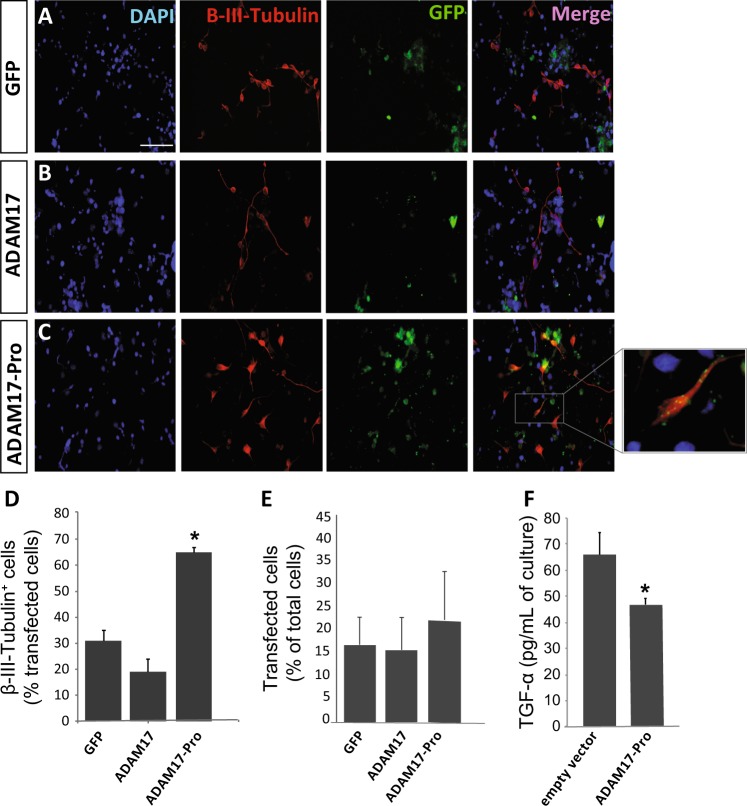
Exogenous expression of the ADAM 17 Pro domain construct ADAM17-Pro increases neuronal differentiation of neural precursors in vitro. **a**–**c** Representative fluorescence microphotographs of SVZ-derived cultured neural precursors that had been transfected with pcDNA3.3 vectors for the expression of: **a** the green fluorescent protein (GFP), **b** a chimera made of the metalloprotease ADAM17 tagged to GFP, or **c** a construct made with the coding region of the ADAM17 pro-domain (ADAM17-Pro) tagged to GFP. Cells were allowed to differentiate for 72 h after transfection and then fixed. Neuronal cells were identified by the immunocytochemical detection of β-III-tubulin (red); transfected cells were recognized by their green fluorescence (GFP), and total nuclei were counterstained with DAPI (blue). Scale bar = 100 µm. **d** Graph represents the percentage of transfected cells that were positive for β-III-tubulin expression. **e** Graph represents the percentage of transfected cells relative to total number of cells. Data are the mean ± S.E.M.; *n* = 3 independent experiments performed in triplicates. **f** ELISA-based analysis of the concentration of TGFα in the culture medium of cells expressing GFP (empty vector) or ADAM17-Pro. A significant decrease in TGFα is observed in ADAM17-Pro expressing cells. Statistical analysis: *p < 0.05 by Student’s *t* test comparing with the control group (GFP) and ADAM17 transfected group

To test this construct in vitro, the coding sequence of ADAM17-Pro was fused to that of GFP, and cloned into a pCDNA3.3 plasmid. Adhered NPC were then transfected with pCDNA3.3-GFP (empty vector), pCDNA3.3-ADAM17-wt-GFP, or pCDNA3.3-ADAM17-Pro-GFP, and were cultured for 72 h in the absence of growth factors, to favor NPC differentiation (Fig. 5). ADAM17-Pro was able to reduce soluble TGFα, a by-product of ADAM17 activity, in the culture medium to almost 60% of control cultures (Fig. 5f). The percentage of GFP^+^ cells expressing the neuronal marker ß-III-tubulin was analyzed in NPC cultures transfected with the different plasmids. Transfection efficiency was similar in all conditions tested (Fig. 5e). However, while 31% and 20% of GFP^+^ cells expressed ß-III-tubulin after transfection with the empty GFP vector (control) or with ADAM17wt, respectively, this percentage significantly increased in cultures transfected with ADAM17-Pro, where 70% cells of GFP^+^ cells expressed ß-III-tubulin (Fig. 5d). These results indicated that overexpression of the dominant-negative ADAM17 mutant in vitro promoted the differentiation of NPC towards a neuronal fate.

### Overexpression of ADAM17-Pro within the injured cortex promotes local generation of neuroblasts and neurons

In order to test the in vivo effects of ADAM17 inhibition within mechanical cortical injuries, a lentiviral vector was designed to overexpress the ADAM17 pro-domain locally in cortical lesions, which also contained the GFP ZS-Green coding region as a reporter. This lentiviral vector allowed for the independent expression of ADAM17-Pro and ZS-Green, as co-translation of both proteins was governed by an IRES element.

**Fig. 6:**
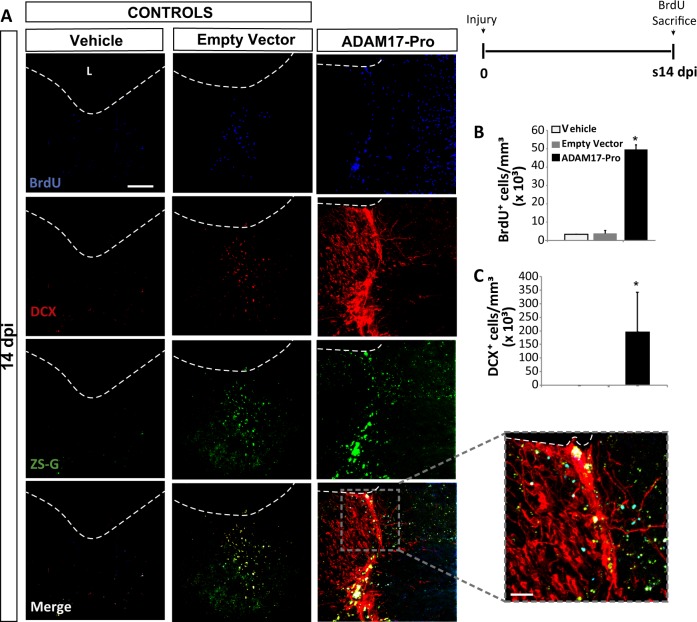
Local inhibition of ADAM17 activity in the injured cortex increases neuroblast number. Mechanical cortical lesions were unilaterally performed in the primary motor cortex of adult mice and locally injected with vehicle, an empty lentiviral vector expressing Zs-Green (both of them as controls) or a lentiviral vector expressing the Pro domain region of ADAM17 (ADAM17-Pro). Animals were sacrificed 14 (s14 dpi), after receiving BrdU injections as explained in the figure. The upper right panel shows the experimental protocol for BrdU injection in s14 dpi mice, which were injected on the day of sacrifice. **a** Fluorescence representative images of the area surrounding cortical lesions in mice showing proliferating BrdU^+^ cells (blue), the green fluorescent protein ZS-Green, and the early neuronal marker DCX (red). **b** Quantification of BrdU^+^ cells/mm^3^ in the perilesional area of the indicated animal groups. **c** Quantification of DCX^+^ cells/mm^3^ in the perilesional area of the indicated animal groups. Dotted lines delineate cortical lesion borders. Scale bar = 50 µm. Data shown are the mean ± S.E.M.; *n* = 3–6 animals per group. Statistical analysis: ANOVA and Bonferroni posttest, *p < 0.05 when compared with rest of groups. L lesion

Controlled mechanical cortical lesions were performed in mice and, immediately after, the injury was injected with either a lentiviral vector expressing only ZS-Green (empty vector), or with a lentiviral vector expressing both ZS-Green and ADAM17-Pro. A second type of control lesions were obtained by injecting vehicle alone. Then, some mice were sacrificed 14 days after the lesion/transduction procedure (s14 dpi), which received three BrdU injections on the day of sacrifice, whereas another group was sacrificed 28 dpi (s14+14 dpi), after receiving BrdU injections every 48 h from day 1 to day 14 (Fig. 6).

**Fig. 7:**
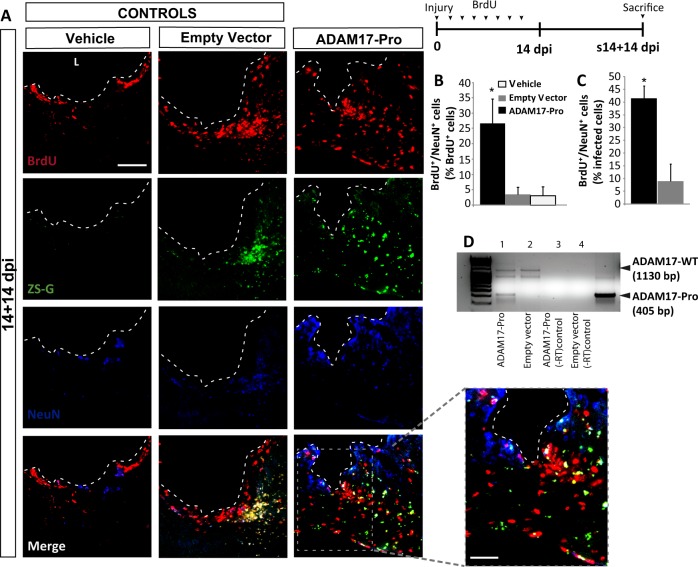
Local inhibition of ADAM17 activity in the injured cortex promotes the differentiation of neuroblast to mature neurons. Mechanical cortical lesions were unilaterally performed in the primary motor cortex of adult mice and locally injected with vehicle, an empty vector (both of them as controls) or Pro domain region of ADAM17 (ADAM17-Pro). Animals were sacrificed 28 days post-injury (s14+14 dpi), after receiving BrdU injections as explained in the figure. The upper right panel shows the experimental protocol for BrdU injection for s14+14 dpi mice, which were injected every 2 days for 14 days, and then sacrificed 14 days later, this is, 28 dpi. **a** Fluorescence microscopy images of the perilesional area showing proliferating BrdU^+^ cells (red), the green fluorescent protein ZS-Green, and the mature neuron marker NeuN (blue). **b** Graph shows the percentage of BrdU^+^ cells that coexpressed NeuN in the perilesional area. **c** Percentage of infected cells (expressing ZS-Green) that co-expressed BrdU and NeuN. Dotted lines delineate cortical lesion borders. **d** Image of an agarose electrophoresis gel showing the RT-PCR product of injury homogenates to detect ADAM17-Pro mutant in mice transduced with the ADAM17-Pro lentiviral vector (ADAM17-Pro); line 1 or the empty lentiviral vector (ZS-Green) line 2. −RT controls in lines 3 and 4. Scale bar = 100 and 50 µm in the high magnification picture. Data shown are the mean ± S.E.M.; *n* = 3–6 animals per group. Statistical analysis: ANOVA and Bonferroni posttest, *p < 0.05 when compared with rest of groups. L lesion

Transduction efficiency measured as the number of ZS-Green^+^ cells/mm^3^ was similar in mice injected with the empty (Zs-Green) lentiviral vector and the ADAM17-pro-ZsGreen lentiviral vector (approximately, 50 × 10^3^ cells/mm^3^), indicating that both vectors equally transduced the injured area. Compared to controls (empty vector- and vehicle-injected mice) those s14 mice that were injected with ADAM17-Pro showed a significant increase on the number of BrdU^+^ cells within the lesion (Fig. 6a, b). This increase on perilesional BrdU^+^ cells induced by specific ADAM17 knockdown was much larger than that induced by broad metalloprotease inhibition with GM6001 (compare Figs. 1d and 6b). In addition, lesions transduced with ADAM17-Pro showed a significant amount of DCX^+^ cells, and 5% of all BrdU^+^ cells were DCX^+^ (data not shown), whereas no DCX^+^ cells were found in lesions injected with the empty vector or with vehicle (Fig. 6a, c). Moreover, the DCX^+^ cells observed within the lesion after ADAM17-Pro overexpression showed a highly differentiated morphology, with clear and prolonged neuritic projections (Fig. 6a). Analysis of s14+14 dpi lesions showed co-localization of BrdU and NeuN in all animal groups (vehicle-, empty vector-, and ADAM17-Pro injected mice), probably due to the intensive BrdU-injection protocol in these s14+14 dpi mice. However, a 10-fold increase in the percentage of newly-formed neurons (BrdU^+^/NeuN^+^) was observed in lesions infected with ADAM17-Pro, when compared to control lesions (Fig. 7a–c). In addition, the ADAM17-Pro mRNA was detected in the lesion by RT-PCR (Fig. 7d). These results indicated that ADAM17-Pro induced the generation of new neurons within the injured area.

### Overexpression of ADAM17-Pro within the injured cortex promotes the generation of cholinergic neurons

**Fig. 8:**
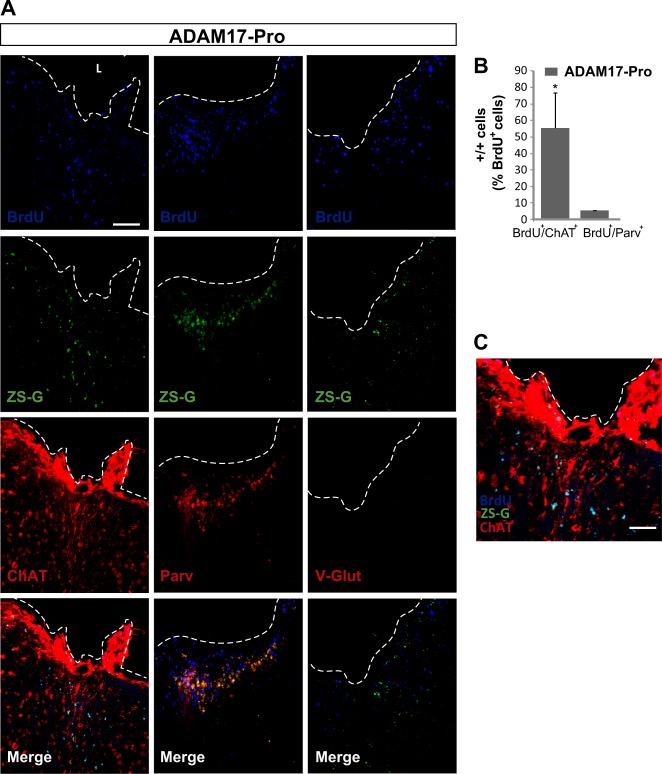
Local inhibition of ADAM17 activity in the injured cortex promotes the generation of cholinergic neurons. Mechanical cortical lesions were unilaterally performed in the primary motor cortex of adult mice and, immediately, the lesion was locally injected with the construct expressing the Pro domain region of ADAM17 (ADAM17-Pro). Mice were then intraperitoneally injected with BrdU every 2 days during the first 14 days, and were sacrificed on day 28 post injury. **a** Representative images of the area surrounding cortical lesions showing proliferating BrdU^+^ cells, the cholinergic neuron marker ChAT, the GABAergic neuron marker parvalbumin, and the glutamatergic neuron marker V-Glut. Scale bar = 100 µm. **b** Graph shows the percentage of BrdU^+^ cells that coexpressed ChAT or Parvalbumin. Glutamatergic neurons were not found. **c** Representative image of merge in panel A (zoom in) left column showing double-labeled cells (BrdU^+^/ChAT^+^) in the perilesional area. Scale bar = 50 µm. Dotted lines delineate cortical lesion borders. L lesion

The phenotype of the newly generated neurons was further characterized in ADAM17-Pro-infected s14+14 dpi mice, by using the marker parvalbumin to detect GABAergic neurons, choline acetyltransferase (ChAT) to detect cholinergic neurons, and VGlut to detect glutamatergic neurons. Almost 50% of BrdU^+^ cells were ChAT^+^ within the injured area, whereas nearly 5% co-localized with parvalbumin and no VGlut labeling was detected (Fig. 8). These results indicated that, by knocking down ADAM17 activity in primary motor cortex lesions, the majority of the new neurons formed are cholinergic.

## Discussion

Based on our previous work on the role of ADAM17 inhibition in mediating neuroblast generation in vitro^14^, it seemed reasonable to hypothesize that the specific inhibition of ADAM17 in vivo in brain lesions might create a neurogenic environment facilitating the generation of new neurons. The results presented here show that a gliogenic environment is generated in non-treated cortical brain injuries, which prevents both the generation of neuroblasts and their migration from the SVZ to the injury, thus impairing neuronal renewal within the damaged area. In this environment, pan-metalloprotease inhibition not only promoted the generation of new neurons, but it also allowed survival and migration of neuroblasts from neurogenic niches, likely the adjacent SVZ. In addition, a more potent neurogenic effect could be observed in injuries in which the specific enzymatic activity of ADAM17 had been blunted.

### Local pan-metalloprotease inhibition in injured cortex creates a neurogenic environment

Previous studies describe a neurogenic response to lesions in the cerebral cortex or in the spinal cord^34–38^. Most of these studies analyze the response of DG and SVZ to an injury rather than focusing on the injured-tissue response. Our work characterizes the response of the primary motor cortex to a focal injury at different time points: 3, 7, 14, and 28 dpi, as well as the response of the DG and SVZ to this specific injury.

In the absence of any treatment, an acute tissue response to the injury was observed, characterized by a fast proliferation of undifferentiated progenitors during the first 7 days, most of which finally differentiated into astrocytes, but never to neuroblasts. A glial scar was rapidly formed by the activation of pre-existing astrocytes present within the injured area, although further addition of astrocytes generated de novo from NPC might likely be contributing to it. Together, these results show that a cortical injury constitutes a gliogenic/non-neurogenic environment, which favors glial differentiation of NPC and prevents either neuroblast formation or their integration/survival after migration from neurogenic niches.

Inside this gliogenic context, local inhibition of metalloprotease activity with GM6001 (50 μM) decreased glial differentiation of neural progenitors, and favored neuroblast formation and differentiation to mature neurons.

### Local metalloprotease inhibition in cortical lesions promotes migration of cells from the SVZ to the injury

Proliferative (BrdU^+^) cells appeared throughout the peri-lesional area in response to cortical injury, and their number increased significantly after chronic treatment with GM6001. Previous results have demonstrated that migration of cells from the SVZ towards a cortical injury does not easily occur^7^. We were able to identify neuroblasts migrating from the SVZ towards the lesion site through the corpus callosum, which never reached the lesion unless the metalloprotease inhibitor GM6001 was present. Our work further demonstrated that at least part of the BrdU^+^ cells found in GM6001-treated lesions had come from distant neurogenic niches, likely the SVZ, while non-treated lesions were devoid of these cells. Once at the lesion site, the phenotype of migrating cells varied from undifferentiated progenitors, to glial cells or neuroblasts. The mechanism by which metalloprotease inhibition exerted this effect remains unknown. It could be the result of increased chemoattraction of cells migrating from neurogenic areas towards the lesion; alternatively, GM6001 could be altering the pattern of proinflammatory-cytokine secretion within the injury, to increase the survival of the BrdU^+^ cells arriving at the lesion site. In relation to this, the metalloprotease ADAM17/TACE is responsible for the shedding and activation of the proinflammatory molecule tumor necrosis factor alpha, and its inhibition could be beneficial for cell survival within the lesion^39^.

### The neurogenic effect of specific ADAM17 inhibition surpasses that of broad metalloprotease inhibition

Our work finally addressed the question of whether specific inhibition of the gliogenic pathway ADAM17-TGFα-EGFR^14,40^ in lesions promoted neurogenesis. To inhibit ADAM17 we overexpressed the ADAM17 pro-domain (ADAM17-Pro), a potent inhibitor of the catalytic domain of this metalloprotease^26,27^. The pro-domain is located at the N-terminal part of the protein, and needs to be removed for the catalytic domain to become active; ADAM17-Pro overexpression prevents the catalytic domain from accessing a native functional state^41,42^.

Thus, we have used this previously described pro domain construct (ADAM17-Pro) to inhibit ADAM17 activity in vitro and in vivo, and to test whether this inhibition promoted neuronal differentiation. Although it is possible that ADAM17-Pro could inhibit other ADAMs, reports show that it may inhibit ADAM10 but not other ADAMs^27^. We do not think this dual inhibition was critical in our study since only ADAM17 and not ADAM10 is overexpressed in injured tissue, and inhibition of ADAM10 in vitro does not exert any effect on neuronal differentiation^14^.

Ablation of ADAM17 activity by ADAM17-Pro increased cell proliferation within the injury as well as the generation of neuroblasts, and its effect was much more pronounced than that exerted by the general metalloprotease inhibitor. Moreover, the neuroblasts generated in the presence of ADAM17-Pro showed a more complex and differentiated morphology than those generated in the presence of GM6001. Additionally, ADAM17-Pro induced the differentiation of these neuroblasts into NeuN^+^ mature neurons. We partially identified the phenotype of new neurons as ∼55% ChAT^+^ and 5% GABAergic; no new glutamatergic neurons were found. These results agree with a recent study, which shows that reactive astrocytes generated following a cerebral medial artery occlusion transdifferentiate into progenitors that produce cholinergic and GABAergic neurons in the striatum^43^. ChAT^+^ cells might further facilitate neurogenesis within the injury, since, in the SVZ, acetylcholine released by subependymal cholinergic neurons promotes neurogenic proliferation^44^.

In synthesis, our work demonstrates that inhibition of ADAM17 in cortical lesions favors neuronal formation by promoting the differentiation of progenitors and by facilitating migration of neuroblasts from the SVZ towards the injured area. We show that ADAM17 might be a therapeutic target to develop strategies aimed to repair the injured brain.

## Electronic supplementary material

Figure S1

Figure S2

Figure S3

Figure S4
